# Do commodity assets hedge uncertainties? What we learn from the recent turbulence period?

**DOI:** 10.1007/s10479-022-04876-0

**Published:** 2022-09-12

**Authors:** Md. Bokhtiar Hasan, Md. Naiem Hossain, Juha Junttila, Gazi Salah Uddin, Mustafa Raza Rabbani

**Affiliations:** 1 Department of Finance and Banking, Islamic University, Kushtia, 7003 Bangladesh; 2 School of Business and Economics, University of Jyväskylä, Jyväskylä, Finland; 3 Department of Management and Engineering, Linköping University, Linköping, Sweden; 4 Department of Economics and Finance, College of Business Administration, University of Bahrain, Sakhir, Bahrain

**Keywords:** Uncertainties, COVID-19, Commodities, Safe-haven, DCC-GARCH, Quantile-on-quantile, C22, C58, G11, G15

## Abstract

This study analyses the impact of different uncertainties on commodity markets to assess commodity markets' hedging or safe-haven properties. Using time-varying dynamic conditional correlation and wavelet-based Quantile-on-Quantile regression models, our findings show that, both before and during the COVID-19 crisis, soybeans and clean energy stocks offer strong safe-haven opportunities against cryptocurrency price uncertainty and geopolitical risks (GPR). Soybean markets weakly hedge cryptocurrency policy uncertainty, US economic policy uncertainty, and crude oil volatility. In addition, GSCI commodity and crude oil also offer a weak safe-haven property against cryptocurrency uncertainties and GPR. Consistent with earlier studies, our findings indicate that safe-haven traits can alter across frequencies and quantiles. Our findings have significant implications for investors and regulators in hedging and making proper decisions, respectively, under diverse uncertain circumstances.

## Introduction

Investors are generally concerned about various market-related risks and uncertainties when investing their funds in financial and commodity markets (Naeem et al., 2021; Zhang & Yan, 2020). As a result, they search for investable assets that provide hedging opportunities against threats posed by financial markets, adverse economic policy changes, and financial crises such as the 2008 global financial crisis (GFC), the COVID-19 pandemic, etc. The risk-averse investors may suffer from indecisive investments because the global stock markets have responded strongly to growing global risks and fluctuating inter-market connections (Zhang et al., 2020). If the list of diversifiable assets is widened, it will aid investors in making investment decisions more adaptively in the face of uncertainties and crises.

We consider and analyze five vital commodity market assets: the general Goldman Sachs Commodity Index (GSCI), West Texas Intermediate (WTI) crude oil, natural gas, soybeans, and clean energy (CE) stocks to reveal if they can give any hedging possibility against various global uncertainties such as the cryptocurrency policy and price uncertainty (UCRY Policy and Price) indexes, the US economic policy uncertainty (USEPU) index, the Chicago Board Options Exchange (CBOE) volatility index (VIX), the CBOE crude oil volatility index (OVX), and the geopolitical risk (GPR) index.

Commodities are critical to the global economy as they are used as inputs in producing various goods (Chevallier & Ielpo, 2013). Therefore, the commodity market demand/supply shocks due to risk and changing uncertainty conditions can cause price volatility, which can have severe ramifications for the industries that use these commodities (Larosei and Mally, 2016). Although commodities are not financial assets, their prices may have a connection with financial asset prices, as the commodity markets are dependent on many macroeconomic factors, including economic policies (Roache & Rossi, 2009, Larosei and Mally, 2016). Many investors invest in commodities not only for the purpose of acquiring ownership but also for the benefit of diversification or hedging for their portfolios (Larosei and Mally, 2016). Therefore, equity investors in specific industries where these commodities are used in manufacturing must be aware of commodity price fluctuations. Also, when designing policies that need real-time monitoring, a real-time assessment of commodities' hedge or safe-haven capabilities is crucial. As a result, exploring various factors that may impact the commodity markets is crucial to investors, government agencies, and other stakeholders, which leads us to consider the commodity market. Furthermore, because some of the aforementioned commodity market assets have been demonstrated to have a negative influence on the stock and bond markets, they may emerge as an alternative investment tool, bolstering the long-term asset diversification strategies (Gorton & Rouwenhorst, 2006; Ji et al., 2020).

Although there is also some empirical evidence for the influence of various uncertainty indices on commodity markets, the evidence for the effect of cryptocurrency uncertainty indices has yet to be documented. However, in recent years, the cryptocurrency market has experienced unprecedented growth. According to CoinGecko, 2021 was a milestone year for the cryptocurrency market, with more than $3 trillion in market capitalization (Hart, 2021). In 2021, investors worldwide invested over $30 billion in cryptocurrencies, which is more than all previous years combined (Dailey, 2021). As a result, individuals globally have embraced cryptocurrencies as a means of transaction. Noticing the trend, a growing number of well-known organizations, including Microsoft, Tesla, Amazon, Visa, PayPal, Starbucks, and others, have either embraced or are planning to accept cryptocurrencies as a payment form. Hence, in addition to the fiat currency, cryptocurrencies as a medium of exchange are increasingly becoming a vital part of the economy worldwide. Since the volatility of fiat currency exchange rates of several major currencies (e.g., USD, GBP) has empirically had a significant effect on commodity prices (Arezki et al., 2014; Rossi, 2013; Zhang et al., 2016), the volatility in cryptocurrency markets is likely to influence commodity prices as well, which is still unexplored.

Moreover, cryptocurrencies are now termed crypto commodities, and hence, they have a connection with other commodity assets (Mo et al., 2022). Accordingly, uncertainty stemming from the crypto market in terms of both policy and price may also have an influence on the commodity markets. Scant previous research has recently focused on this issue. For instance, Hassan et al. (2021) find a significant impact of both UCRY Policy and Price on metal commodity markets. Similarly, Elsayed et al. (2022) reveal that UCRY index changes spill over strongly on gold markets. According to Yin et al. (2021), the long-term volatility of cryptocurrency markets has a considerable impact on the oil market. Bejaoui et al. (2022) also discover a strong correlation between Bitcoin and commodity assets such as crude oil and natural gas. Hassan et al. (2022) recently examined the relationship between the cryptocurrency environmental attention index (ICEA) and three asset classes, including commodity markets, and found that the commodity assets, such as soybeans, had a positive correlation with it. Hence, it appears that the cryptocurrency market and its uncertainty are linked to the commodity markets, so the crypto market’s uncertainty is likely to be connected with other commodity markets, which requires investigation for more comprehension. All of these previous findings have motivated us to include the cryptocurrency uncertainty measures in this study.

The UCRY Policy and Price indices,^1^ introduced by Lucey et al. (2022), are recent innovations in this respect and have already been found to be significant in forecasting the risks that arise from the cryptocurrency marketplaces. Considering the most recent events, i.e., the COVID-19 pandemic, Lucey et al. (2022) note that the cryptocurrency uncertainty indices may move differently from other risk and uncertainty indicators, indicating hedging opportunities. Thus, we explore four other uncertainty indices to verify this statement—i.e., USEPU, VIX, OVX, and GPR. Using this approach, we can determine the influence of uncertainty indices on commodity assets, identify safe-haven assets against these uncertainties throughout the sample period and COVID-19 crises, and compare the cryptocurrency uncertainties’ impacts with those of the other four uncertainty indicators.

The existing literature on the impact of uncertainty indicators has increased considerably and claims that several uncertainty indicators have a significant influence on financial markets. In contrast, research on cryptocurrency policy and price uncertainty is still scarce. To the best of our knowledge, only three studies (Elsayed et al., 2022; Hasan et al., 2022; Hassan et al., 2021) have investigated the role of the UCRY Policy and Price indices on different asset classes. Hasan et al. (2022) test the potential and conventional safe-haven assets (gold, Bitcoin, US Dollar, DJ Islamic, Sukuk, and WTI) against UCRY Policy. Conversely, Hassan et al. (2021) and Elsayed et al. (2022) use both UCRY Policy and Price to examine the precious metals’ safe haven properties: gold, silver, platinum, and palladium. Our study differs from the studies above by focusing on five different commodity assets—GSCI, WTI crude oil, natural gas, soybeans, and CE—to see their hedging opportunities against six different uncertainties, including UCRY Policy and Price. Also, our study diverges from these studies from a methodological standpoint.

The studies mentioned above leave out some crucial points, such as whether the cryptocurrency uncertainty indices behave differently with respect to the asset returns compared to the other types of uncertainty indices, as there are some differences in the construction and basic ideas between the cryptocurrency and other uncertainty indices (Lucey et al., 2022). Second, determining the assets' safe-haven potential during the COVID-19 pandemic is crucial since safe-haven is especially relevant during market downturns and crises (Baur & Lucey, 2010). In light of the preceding, this research aims to address the following unexplored research issues. First, how are the commodity assets linked to the various uncertainties? Second, do the commodity assets provide hedging benefits in the face of uncertainty? Third, do commodity assets' hedging attributes alter across time, especially before and during the COVID-19 crises? Finally, do the cryptocurrency uncertainty indicators differ from the other uncertainty indices (USEPU, VIX, OVX, and GPR) in terms of their links to the commodity markets?

Employing the DCC-GJR-GARCH (1,1) and wavelet-based Quantile-on-Quantile (QQ) regression models from December 30, 2013, to April 22, 2021, our study finds that only soybean and CE stock markets have strong safe-haven properties against UCRY Price and GPR, even during the COVID-19 crisis. However, our study also finds weak safe-haven behavior in the GSCI commodity and WTI against both the cryptocurrency uncertainty indices and GPR. Finally, we assert that the UCRY indices impact commodities differently from other uncertainty indicators, excluding GPR.

Our study contributes to the existing literature from five viewpoints. First, unlike earlier studies, this study considers five vital commodity assets to assess the probable effects of six popular uncertainty indices and to detect these assets' hedging and safe-haven potential in the face of such uncertainties. Second, we mainly discuss the characteristics of the volatility and dynamic associations between the commodity assets and uncertainty factors before and during the COVID-19 crisis. Third, our findings show that the soybean market has a higher risk hedging potential than the others when it comes to UCRY Policy and Price, USEPU, OVX, and GPR, and thus can be utilized to safeguard investors' portfolios from financial losses caused by these uncertainties. Fourth, according to our understanding, following Lucey et al. (2022), we are the first to confirm that the UCRY Policy and Price influence assets differently than the other uncertainty indices. Finally, we extend the list of alternative investment assets to provide hedging benefits, especially during market downturns.

The remaining structure of the paper is as follows. Section 2 reviews the related literature; Sect. 3 explains the data and summary statistics; Sect. 4 provides methodology; Sect. 5 represents, analyzes, and discusses the findings of this study; and finally, Sect. 6 concludes the study.

## Literature review

Financial markets are adversely affected by many types of uncertainty, exacerbated by financial crises in recent decades—for example, the Asian financial crisis in 1997, GFC 2008, and COVID-19. These uncertainties and crises cause investors to suffer significant losses, prompting them to look for alternative assets with hedging and safe-haven properties. However, prevailing safe-haven assets, such as gold, Treasury bonds, foreign currencies, and Bitcoin, do not often safeguard investors against financial crises (Hasan et al., 2021a; Shahzad et al., 2019). As a result, recent studies seek safe-haven characteristics in other assets, such as commodity assets (especially the general GSCI index, WTI, and soybeans).

As such, some studies in the extant literature (e.g., Azar & Chopurian, 2018; Bouri et al., 2020; Fernandez, 2019; Hasan et al., 2021a; Shahzad et al., 2019) consider the general GSCI commodity index to assess the safe-haven ability of commodity markets, as well as the spillover effect or relationship with different assets or uncertainty measures. More specifically, Shahzad et al. (2019) and Bouri et al. (2020) reveal that the GSCI commodity index might serve as a weak safe-haven asset against the risks in stock market returns. Similarly, Azar and Chopurian (2018) show that the commodity index serves as a risk diversifier in G7 countries against market volatility during some circumstances. Moreover, Badshah et al. (2019) find that the commodity index is positively and significantly linked to the economic policy uncertainty (EPU) index but insignificantly associated with the stock market uncertainty (VIX) index. However, Hasan et al. (2021a) discovered no safe-haven features of the GSCI commodity index for the US stock markets during the GFC 2008 and the subsequent COVID-19 crisis.

The WTI oil market is perhaps the most important and widely studied commodity market. A number of studies have looked at the WTI’s hedging capabilities in the face of various types of uncertainty but with conflicting results. Arunanondchai et al. (2020), Ji et al. (2020), and Tarchella and Dhaoui (2021), for example, trace the WTI’s hedging function, particularly during the COVID-19 crisis. Dahl et al. (2020), Elie et al. (2019), Jin et al. (2019), and Hasan et al., (2021a, 2022), on the other hand, investigate the safe-haven properties of WTI and find none. Likewise, Hasan et al. (2021a) reveal the inconsistent result about WTI's safe-haven role in the GFC 2008 and COVID-19 for the US stock markets. However, the findings of Lei et al. (2019) are mixed. They show that before the financial crises, EPU has a considerable negative influence on WTI, but the coefficient turns positive after the crises. Similarly, Aloui et al. (2016) find a negative relationship between uncertainties (EPU and equity market uncertainty (EMU)) and crude oil returns during normal times but a positive relationship during financial crises. Zhang and Yan (2020) unveil that the WTI returns are negatively affected by different EPU indices. However, Antonakakis et al. (2017) and Qin et al. (2020) document a negative impact of geopolitical risk on WTI.

Despite the scarcity of research on the subject, natural gas—another crucial commodity asset—can provide a useful hedging strategy. Arunanondchai et al. (2020) look into exchange-traded funds (ETFs) to hedge energy commodity tail risk. They show that ETFs offer greater downside risk protection in the natural gas markets. Badshah et al. (2019) find natural gas a strong shelter against EPU. Zhang et al. (2017) find that the stock market volatility (VIX and VSTOXX) has a spillover effect on WTI and natural gas markets.

Another vital commodity market related to the emerging asset class, i.e., the soybeans, has also been evaluated as a prospective safe haven from other angles against market uncertainty in several earlier studies (e.g., Badshah et al., 2019; Dahl et al., 2020; Ji et al., 2020). According to Ji et al. (2020), soybeans were a powerful risk diversifier and safe-haven asset during COVID-19. Chang and Su (2010) also find strong volatility spillovers across crude oil, corn, and soybean markets.

Several studies have looked into CE equities, which may be regarded as one of the most sophisticated energy market assets due to current worries about climate change (e.g., Albulescu et al., 2019; Dutta, 2017; Dutta et al., 2020; Pham, 2019). However, in previous studies, only a few have identified CE's hedging ability. For example, at the lower tails of the return distribution, Albulescu et al. (2019) ratify the CE stock market as a powerful risk diversifier in the case of severe bull market events. Pham (2019) documents a heterogeneous association between oil price and CE stocks and finds evidence of the hedging effectiveness of CE investments against the oil price shocks. Moreover, Dutta (2017) finds that the CE stock market returns are highly vulnerable to the WTI volatility index (OVX). However, in the case of energy sector volatility (VXXLE), Dutta et al. (2020) uncovered a negative impact on CE stocks during high and low volatility regimes.

From the above literature, some issues stand out. First, past findings on the impact of various uncertainty measures on chosen commodities are equivocal. As a result, there is no consensus on their hedging efficacy. Second, among the selected commodities, only the WTI market has been widely researched. Still, few have considered the GSCI commodity, natural gas, soybeans, and CE stock returns to trace their hedging potential against various kinds of uncertainty, including USEPU, VIX, OVX, and GPR. Furthermore, the recently introduced cryptocurrency uncertainty (UCRY Policy and UCRY Price) measures are entirely missed in this context. Therefore, the safe-haven properties of alternative financial assets against these cryptocurrency uncertainty indices are crucial to explore for investors' benefit. Third, to the best of our knowledge, no single study has yet considered six uncertainty indices (i.e., UCRY Policy, UCRY Price, USEPU, VIX, OVX, and GPR) simultaneously to find hedging tools from commodity markets. This study, however, intends to bridge the literature gaps mentioned above.

## Data and preliminary analysis

In the empirical regression models used in this study, the dependent variables are the five key commodity assets’ returns, whereas the independent variables are the changes of six familiar uncertainty indices (see Table 1 for more details). The commodity assets are selected based on the hedging potentials after reviewing the literature. There are 382 weekly observations in our sample, which runs from December 30, 2013, to April 22, 2021. We choose the starting date and data frequency based on the availability of cryptocurrency market uncertainty (UCRY) indices, and the end of our data sample period is determined by the data availability of the US economic policy uncertainty index.^2^ All the price level observations are transformed into logarithmic returns (value changes in case of the uncertainty measures) to assure the stationarity of the analyzed time-series data in the empirical analyses.

**Table 1:** Details of variables and data sources

Dimensions	Name of variables	Abbreviation	Sources
Commodity markets	S&P GSCI Commodity Index	GSCI	www.investing.com
	S&P WTI Crude Oil Index	WTI	
	S&P GSCI Natural Gas Index	Natural gas	www.spglobal.com
	Dow Jones Commodity Index-Soybeans	Soybeans	
	S&P Global Clean Energy Index	CE	
Uncertainty indices	Cryptocurrency Policy Uncertainty	UCRY Policy	https://sites.google.com/view/cryptocurrency-indices/home?authuser=0
	Cryptocurrency Price Uncertainty	UCRY Price	
	US Economic Policy Uncertainty	USEPU	www.policyuncertainty.com
	CBOE Volatility Index	VIX	www.investing.com
	CBOE Crude Oil Volatility Index	OVX	
	Geopolitical Risk Index	GPR	www.matteoiacoviello.com/gpr.htm

GSCI, WTI, and CBOE stand for Goldman Sachs Commodity Index, West Texas Intermediate, and Chicago Board Options Exchange, respectively

**Fig. 1:**
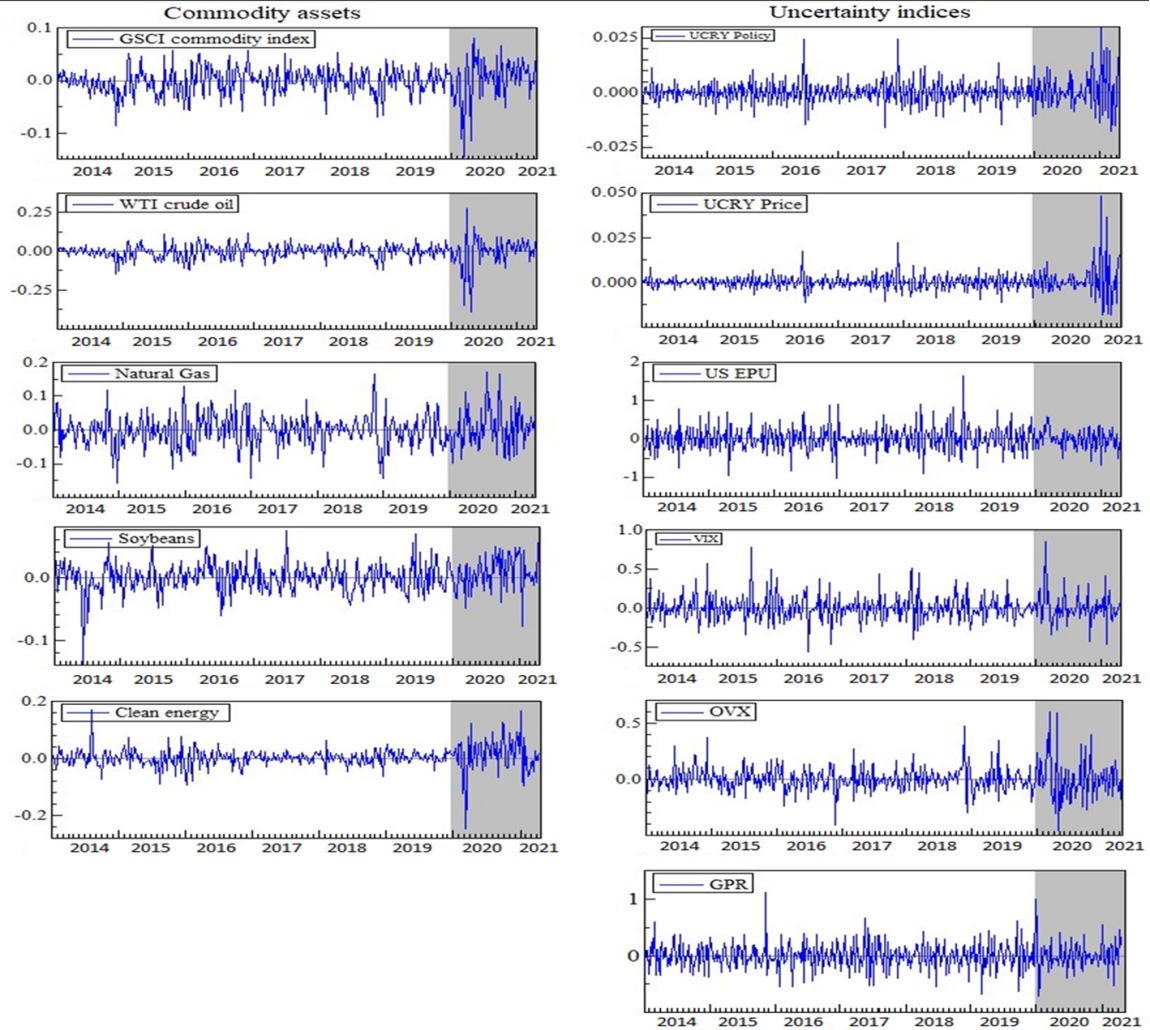
Plots of return series (commodities and uncertainty indices). Note: The shaded areas indicate the returns during COVID-19 (December 31, 2019, to April 22, 2021)

Figure 1 displays the dynamics of the returns of the commodity market assets and log changes of uncertainty indices. All variables in the graphs are strongly time-varying, and their volatility seems to have increased during COVID-19.^3^ During COVID-19, however, as the volatility of uncertainty indices rises, so do the returns on commodity market assets, although not in a comparable way. This could point to the commodity assets' high sensitivity to the shocks in the face of a variety of uncertainty.

The descriptive statistics in panel A of Table 2 report that the CE stocks have the highest mean returns. In contrast, the WTI market has the lowest mean returns and the largest volatility among the commodity assets. Conversely, OVX has the highest mean value among the uncertainty indices, while USEPU has the lowest mean value, with the highest volatility. Small skewness and high kurtosis values for all the commodity return series suggest that the distribution is asymmetric and leptokurtic. Thus, all the return series are non-normally distributed, also evidenced by the Jarque–Bera statistics. However, the Ljung-Box (Qs-20) results confirm that our time series are free from autocorrelation issues. Furthermore, the data has no stationarity issues, indicated by the augmented Dickey-Fuller (ADF) and Phillips and Parron (PP) tests.

**Table 2:** Summary statistics

	ΔlnGSCI	ΔlnWTI	ΔlnNatural gas	ΔlnSoybeans	ΔlnCE	ΔlnUCRY Policy	ΔlnUCRY Price	ΔlnUSEPU	ΔlnVIX	ΔlnOVX	ΔlnGPR
*Panel A: Descriptive statistics, autocorrelation, and unit root tests*											
Mean	− 0.001	− 0.004	− 0.001	0.001	0.002	0.000	0.001	− 0.003	0.001	0.002	0.001
Max	0.081	0.276	0.227	0.074	0.169	0.023	0.038	1.635	0.854	0.600	1.125
Min	− 0.145	− 0.390	− 0.165	− 0.138	− 0.246	− 0.018	− 0.018	− 1.041	− 0.556	− 0.454	− 0.723
Std. Dev	0.029	0.058	0.052	0.024	0.039	0.005	0.005	0.330	0.170	0.125	0.244
Skewness	− 0.877	− 1.408	0.184	− 0.425	− 0.839	0.729	2.170	0.359	0.883	0.832	0.330
Kurtosis	6.090	12.681	4.489	5.813	11.820	6.459	20.288	4.669	6.155	6.773	4.466
Jarque−Bera	200.960*	1618.202*	37.481*	137.425*	1283.126*	224.327*	5056.778*	52.538*	208.068*	270.662*	41.044*
Qs(20)	39.377*	53.109*	31.006**	42.069*	33.178**	71.747*	74.382*	64.399*	33.729**	36.371**	48.470*
ADF	− 16.092*	− 5.864*	− 17.330*	− 17.175*	− 11.383*	− 15.597*	− 14.136*	− 15.716*	− 13.966*	− 20.981*	− 12.275*
PP	− 16.218*	− 15.666*	− 17.209*	− 17.168*	− 18.032*	− 36.083*	− 36.169*	− 34.439*	− 26.755*	− 21.008*	− 128.203*
*Panel B: Correlation matrix*											
ΔlnGSCI	1.000										
ΔlnWTI	0.894*	1.000									
ΔlnNatural gas	0.196*	0.121**	1.000								
ΔlnSoybeans	0.229*	0.081	0.097***	1.000							
ΔlnCE	0.421*	0.329*	0.079	0.149*	1.000						
ΔlnUCRY Policy	0.020	0.039	− 0.014	0.011	− 0.053	1.000					
ΔlnUCRY Price	0.027	0.034	0.018	0.036	0.014	0.887*	1.000				
ΔlnUSEPU	− 0.057**	− 0.065**	− 0.002	− 0.025	− 0.082***	0.093***	0.034	1.000			
ΔlnVIX	− 0.336*	− 0.288*	− 0.056	− 0.123**	− 0.465*	0.112**	0.112**	0.070	1.000		
ΔlnOVX	− 0.603*	− 0.573*	− 0.109**	− 0.131**	− 0.408*	0.073	0.063	0.096***	0.425*	1.000	
ΔlnGPR	0.023	0.006	0.067	0.108**	0.063	0.019	0.054	0.071	− 0.016	0.085***	1.000

Qs (20) refers to the results from the Ljung-Box test for autocorrelation. ‘*,’ ‘**,’ and ‘***’ designate the significance levels at 1%, 5%, and 10% risk levels, respectively. Δln refers to the natural logarithm returns as the first difference

The correlation matrix (Table 2, Panel B) depicts that the GSCI commodity, WTI, and soybean are positively connected with UCRY Policy and Price but negatively with USEPU, VIX, and OVX. Moreover, the natural gas and CE stocks negatively correlate with UCRY Policy, USEPU, VIX, and OVX, while they are positively correlated only with UCRY Price. Conversely, GPR has a significant positive association only with soybeans.

## Methodology

### Modeling of dynamic conditional correlation

First, we use the dynamic conditional correlation (DCC) model as the primary empirical approach in this study. The DCC estimation, proposed by Engle (2002), has become a prevalent approach to assessing the time-varying correlations between variables in the multivariate conditional correlation framework. The DCC model can address the dimensionality issue by decomposing the conditional covariance matrix, while other multivariate GARCH-models cannot (Ma et al., 2019; Pham, 2019). The DCC approach with the Glosten et al. (1993) (GJR) model is based on the Generalized Autoregressive Conditional Heteroscedasticity (GARCH) representation of the data and is thus named the DCC-GJR-GARCH-model. The asymmetric impacts, in the form of leverage effects, can be addressed using the GJR-GARCH model by referring to high or low volatility for positive or negative shocks, respectively (Al Mamun et al., 2020; Hassan et at., 2021). Thus, this study employs the DCC-GJR-GARCH model^4^ as follows:

1 $${r}_{t}=\mu + \psi {r}_{t-1}+{\mathcal{E}}_{t}, {\mathcal{E}}_{t}={\mathrm{z}}_{t}{h}_{t}, {\mathrm{z}}_{t}\sim N\left(1, 1\right),$$

where ${r}_{t}$ = [${r}_{i,t}, . . ., {r}_{n,t}]$ is the (*n* × *1*) vector of the returns on the analyzed assets. $\mu$ is the vector of the constant terms, and $\psi$ denotes the coefficient vector of the autoregressive terms. ${\mathcal{E}}_{t}=\left[{\mathcal{E}}_{i,t}, \dots .., {\mathcal{E}}_{n,t}\right]$ represents the vector of standardized residuals. To regulate the dynamics of variance, we formulate the conditional volatility from the GJR-GARCH (1, 1) model as follows:

2 $${h}_{i,t}^{2}=\upomega +{\alpha}{\mathcal{E}}_{i-1}^{2}+\beta {\upsigma }_{i-1}^{2}+\Upsilon{\mathcal{E}}_{i-1}^{2}{I}_{t-1},$$

where ${I}_{t-1}=1$ if ${\mathcal{E}}_{t-1}<0$, otherwise ${I}_{t-1}=0$. $\Upsilon$ is the leverage term to capture the asymmetric influence of negative or positive shocks. When $\Upsilon>0$, this indicates that the negative shocks impact more than the positive shocks. The parameters $\upomega$, ${\upalpha  }$, $\beta,$ and $\Upsilon$ in Eq. 2 can assure the stationarity of the conditional volatility process only when the conditions $\upomega >0$, ${\upalpha},\omega ,\beta ,\Upsilon\ge 0,$ and $\Upsilon+\frac{ \alpha  +\beta }{2}<1$ are satisfied.

The diagnostic Ljung-Box-tests (Qs-20) depict that the GJR-GARCH specification with student-t distribution is specified correctly as the residuals are free from autocorrelation effects. Therefore, it is assumed that ${E}_{t-1}\left[{\mathcal{E}}_{t}\right]=0$ and ${E}_{t-1}\left[{\mathcal{E}}_{t}, {\mathcal{E}}_{t-1}^{^{\prime}}\right]={H}_{t}$, where $E\left[\cdot \right]={H}_{t}$ represents the conditional expression at time $t$. However, for the conditional variance–covariance matrix, ${H}_{t}$ can be defined as:

3 $${H}_{t}={D}_{t}^{1/2}{R}_{t}{D}_{t}^{1/2},$$

where ${R}_{t}$ denotes the *n* × *n* time-varying correlation matrix, while the diagonal conditional variance is specified by ${D}_{t}=diag\left({h}_{i,t},\dots .,{h}_{n,t}\right).$ Engle (2002) proposes the right-hand side of Eq. 4 directly instead of ${H}_{t}$ as a dynamic correlation framework:

4 $${R}_{t}=diag{({X}_{t})}^{-1/2}{X}_{t}diag{({X}_{t})}^{-1/2},$$

5 $${X}_{t}=\left(1- \alpha  -\beta \right)K+{\upalpha  \text {diag}}{\left({X}_{t}\right)}^{1/2}{\widehat{\upvarepsilon }}_{i,t-1}{{\widehat{\upvarepsilon }}^{\mathrm{^{\prime}}}}_{i,t-1}diag{\left({X}_{t-1}\right)}^{1/2}+\beta {X}_{t-1},$$

where $K$ expresses the *n* × *n* unconditional covariance matrix for the standardized residuals ${{\widehat{\varepsilon }}^{^{\prime}}}_{i,t}$ and when ${\upalpha}$ and $\beta$ are the non-negative values substantial to ${\upalpha }+\beta <1$, the model is called the DCC-GARCH model.

### Quantile-on-quantile (QQ) approach

As the second empirical approach, we employ the Quantile-on-Quantile (QQ) regression approach proposed by Sim and Zhou (2015). This estimation technique can detect the relationship between the variables at every phase of the conditional distribution. Thus, the QQ model yields a comprehensive view of the dependence. This study employs the non-parametric QQ model to assess how chosen six uncertainty indices’ data series quantiles impact the conditional quantiles of the five selected commodity asset returns. The relationship can be written as follows:

6 $${RA}_{t}={\beta }^{\theta }\left({X}_{t}\right)+{\varepsilon }_{t}^{\theta },$$

where ${RA}_{t}$ is the representative of the dependent variables’ logarithmic returns. The unobserved interconnected functions between ${RA}_{t}$ and ${X}_{t}$ are presumed by ${\beta }^{\theta }\left(.\right)$, where, ${X}_{t}$ represents the uncertainty indices (independent variables). The association between the $\theta$-quantile of ${RA}_{t}$ (commodity assets) and $\theta$-quantile of ${X}_{t}$, signified by ${X}^{\tau },$ are examined through linearizing the function of ${\beta }^{\theta }\left(.\right)$ by considering a first-order Taylor expansion of ${\beta }^{\theta }\left(.\right)$ around ${X}^{\tau }$, yielding the following illustration:

7 $$ \beta ^{\theta } \left( {X_{t} } \right) \approx \beta ^{\theta } \left( {X^{\tau } } \right) + \beta ^{{\theta {\prime }}} \left( {X^{\tau } } \right)\left( {X_{t}  - X^{\tau } } \right). $$

Following Sim and Zhou (2015), ${\beta }^{\theta }\left({X}^{\tau }\right)$ and $\beta ^{{\theta {\prime }}} \left( {X^{\tau } } \right)$ can be redefined as ${\beta }_{0}\left(\theta ,\tau \right)$ and ${\beta }_{1}\left(\theta ,\tau \right)$, respectively. Thus, we re-write Eq. 7 as follows:

8 $${\beta }^{\theta }\left({X}_{t}\right)\approx {\beta }_{0}\left(\theta ,\tau \right)+{\beta }_{1}\left(\theta ,\tau \right)\left({X}_{t}{-X}^{\tau }\right).$$

Equation 8 is substituted into Eq. 6 and forms Eq. 9 as follows:

9 $${RA}_{t}={\beta }_{0}\left(\theta ,\tau \right)+{\beta }_{1}\left(\theta ,\tau \right)\left({X}_{t}{-X}^{\tau }\right)+{\varepsilon }_{t}^{\theta }.$$

Finally, we follow Reboredo et al. (2017) to decompose the asset return distributions using wavelet decompositions.^5^ Next, we segregate them into three frequencies corresponding to 4–8, 16–32, and 32–64 weeks for short-run, medium-run, and long-run estimations, respectively. While this study intends to unfold the impact of uncertainty exerted from $\tau$-quantile, we use a Gaussian kernel based on a particular bandwidth by weighing the observations of an empirical quantile of uncertainty in the neighborhood.

## Empirical results and analysis

### DCC-GJR-GARCH (1, 1) estimation

This section reports the findings from the DCC-GJR-GARCH (1, 1) estimation in Table 3 (see Tables 5, 6, 7, 8, 9 and 10 for the details of DCC estimations). The parameters of *⍺* (ARCH 1) and *β* (GARCH 1) are positive and significant in all the cases (except for the GSCI commodity index and WTI market with the negative ARCH parameters). The sum of *⍺* and *β* approaches ≤ 1. Moreover, the GJR-leverage effect (Gamma) is significant for all the commodities (except soybeans and CE) and the six uncertainty indices. The results of several diagnostic tests (standardized squared residuals, multivariate Hosking, and Li-McLeod) and information criteria (Akaike, Shibata, and Hannan-Quin) in Panels B and C for all the cases confirm the goodness of fit of the GJR-GARCH model with the student-t distribution that is free from serial correlation. Overall, we observe a mixed impact of several uncertainties on the time-varying correlations of the commodities.

The UCRY Policy positively correlates with all the commodity assets used in this study. Conversely, only the UCRY Policy has a significant positive impact on WTI returns in the short run. In contrast, the long-run effect on CE stock returns is found at the 10% significance level. Conversely, the UCRY Price has a significant and positive impact on the dynamic correlation of WTI in the short run, while a long-run influence on soybeans is found at the 10% and 5% significance levels, respectively. This implies that the WTI price might have spillover from the cryptocurrency uncertainty shocks in the short run. At the same time, both UCRY Policy and Price significantly impact CE stock in both the short and long run. Conversely, the UCRY Price has a significant and positive influence on soybean returns only in the long run. Hence, among others, the CE stock market has a higher impact from cryptocurrency market shocks. Also, the sum of the DCC parameters (*a* and *b)* is less than 1, indicating high volatility clustering between the UCRY Price and Policy and CE stock returns.

The DCC parameters of all the commodity assets except natural gas are positive and significantly correlated with USEPU at the 1% level in the long run. However, USEPU has a positive and significant influence on natural gas in the short run. Moreover, although all the DCC parameters are positive, only the CE stock returns have a significant long-run coefficient with VIX at the 1% level. Similarly, except soybean, all commodities are significantly influenced by OVX in the long run, with a short-run effect only on the GSCI and WTI. However, GPR significantly positively impacts both GSCI and CE stock in the short and long run, while only WTI in the long run. Because of the rising geopolitical events, global energy and oil price volatility have increased, affecting investment decisions, economization, and asset prices (Smales, 2021). Thus, our DCC findings (both *a* and *b* parameters) suggest the investors should be conscious since both UCRY Price and GPR may have a high volatility spillover effect on CE stocks, while OVX and GPR may have on GSCI and WTI. However, our finding regarding the impact of GPR on WTI is consistent with Smales (2021).

**Fig. 2:**
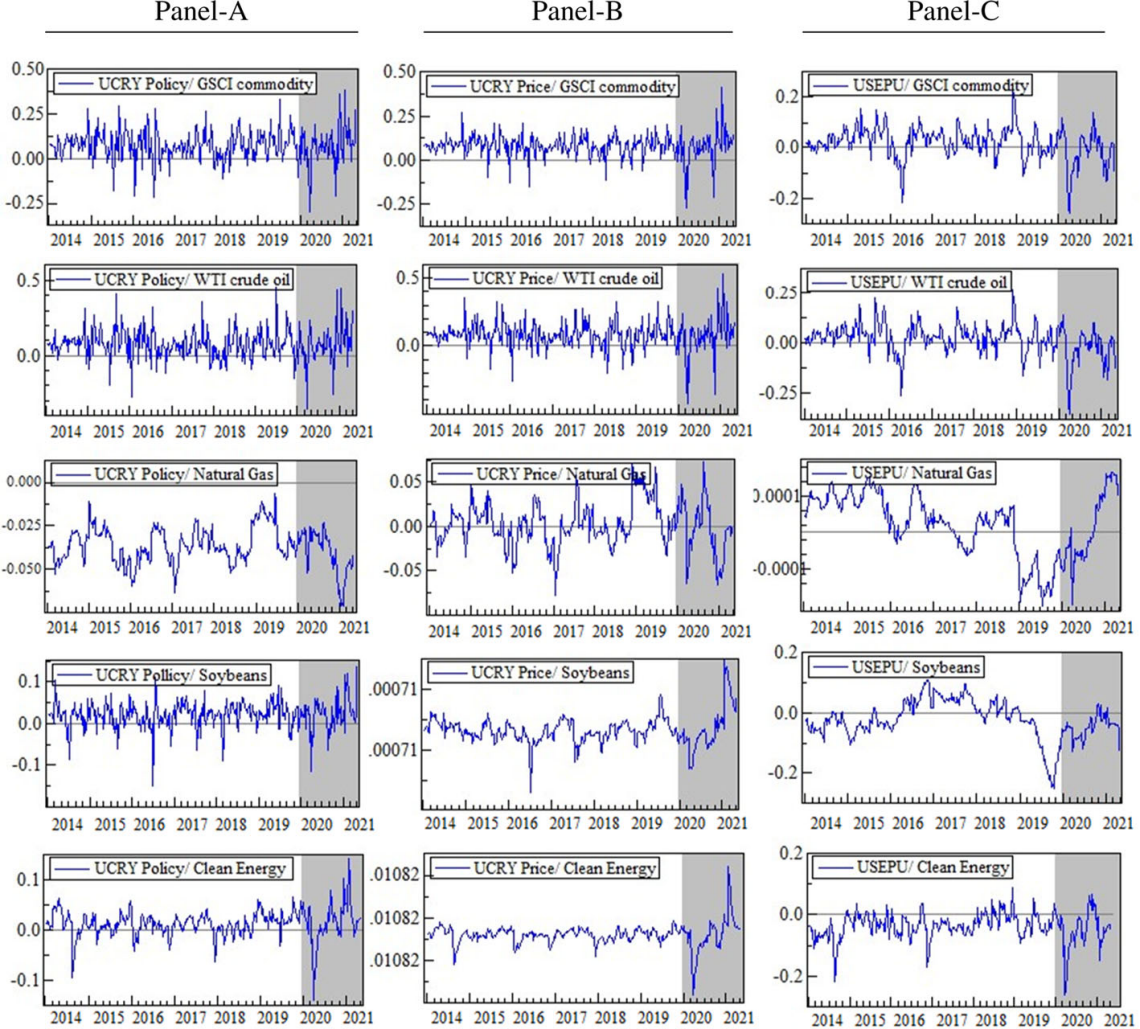
Time-varying conditional correlations (uncertainty indices vs. commodities). Note: The shaded areas indicate the COVID-19 period

**Fig. 3:**
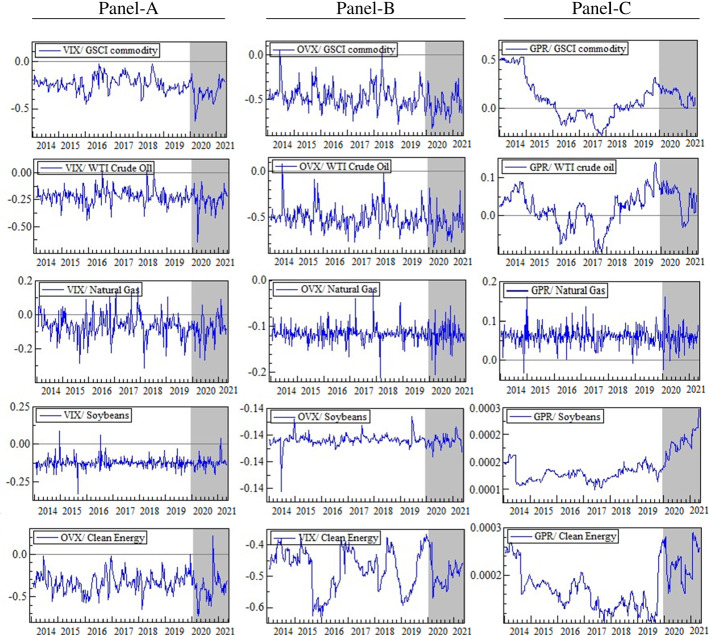
Time-varying conditional correlations (uncertainty indices vs. commodities). Note: The shaded areas indicate the COVID-19 period

Figures 2 and 3 exhibit the time-varying dynamic conditional correlations between uncertainty indices and commodity assets. Interestingly, there appears to be a structural change in the connection of commodity market returns with the financial market uncertainties during the COVID-19 crisis period, indicating a drop in the pairwise correlations during downturns and rising circumstances. For example, Fig. 2 (Panel-B) shows that soybeans and CE stocks have positive and significant correlations with the UCRY Price for the whole period, with a more volatile correlation during the pandemic than during the normal period. These results demonstrate that CE stocks and soybeans yield higher returns when the uncertainty emanates from the cryptocurrency prices, implying that the CE and soybean markets demonstrate strong hedging opportunities against the UCRY Price, which becomes more robust during COVID-19. According to Bloomberg (2021), during COVID-19, Bitcoin prices rose by 300% in 2020 amid speculation in the financial markets where investors hoarded digital currencies due to the lower interest rates; thus, the European central bank cautioned that the Bitcoin investors might lose everything.^6^ This may have escalated the cryptocurrency price uncertainty, evidenced by the steady upward trend in UCRY indices (see Fig. 5). In contrast, soybean prices fell in the first quarter of 2020 due to the COVID-19 hit, then bounced back and continued to rise sharply until mid-2021, attributable to the post-COVID-19 recovery of the global demand (Vos et al., 2022). Realizing the devastating consequences of global climate change due to increased carbon emissions, the entire world has committed to switching to clean energy sources to lower the carbon emissions, leading to the sharp growth in this sector in recent years (Ghabri et al., 2021). These phenomena may cause a positive association between CE and soybeans and UCRY Price. However, our findings partially corroborate the results of Ren and Lucey (2022) and Hassan et al. (2022).

The average correlation (see Table 3) of UCRY Policy and Price (see Fig. 2, Panels A and B) with GSCI and WTI returns is positive but not throughout the sample period. This implies that the UCRY Policy and Price can be hedged marginally by investing in GSCI commodity and WTI markets even during COVID-19. The findings partially contradict Hasan et al. (2022), who showed that UCRY Policy negatively influences WTI. Similarly, soybeans and CE stocks can also marginally hedge the UCRY Policy since they have positive average correlations with UCRY Policy even during the COVID-19 period (see Panel-A). Conversely, the USEPU risks can be partially mitigated by GSCI and WTI (Fig. 2, Panel C), as the average correlations between them are positive (Table 3). The average correlation plunges to negative during the COVID-19 pandemic, suggesting that in times of crisis, the GSCI commodity and WTI may not provide safe-haven benefits against USEPU. Our results confirm the previous findings that the policymakers and investors should always watch economic policy uncertainty, as it may be a significant source of commodity market volatility (Ahmed & Sarkodie, 2021; Sarkodie et al., 2022).

**Table 3:** Results from DCC-GJR-GARCH (1, 1) estimation

Uncertainties	Parameters	ΔlnGSCI	ΔlnWTI	ΔlnNatural gas	ΔlnSoybeans	ΔlnCE
ΔlnUCRY Policy	Avr. Corr	0.077	0.075	− 0.031	0.009	0.003
	Dcc (*a*)	0.091	0.109***	0.000	0.015	0.000*
	Dcc (*b*)	0.287	0.240	0.510	0.428	0.816***
ΔlnUCRY Price	Avr. Corr	0.065	0.056	0.000	0.011	0.014
	Dcc (*a*)	0.080	0.120***	0.003	0.000	0.000*
	Dcc (*b*)	0.307	0.245	0.874	0.748**	0.744***
ΔlnUSEPU	Avr. Corr	0.009	0.018	0.006	− 0.001	− 0.026
	Dcc (*a*)	0.048	0.058	0.000*	0.006	0.028
	Dcc (*b*)	0.747*	0.682*	0.079	0.959*	0.735*
ΔlnVIX	Avr. Corr	− 0.238*	− 0.222*	− 0.062	− 0.125**	− 0.478*
	Dcc (*a*)	0.049	0.061	0.052	0.037	0.022
	Dcc (*b*)	0.814	0.484	0.4857	0.000	0.925*
ΔlnOVX	Avr. Corr	− 0.486*	− 0.520*	− 0.102**	− 0.136**	− 0.333*
	Dcc (*a*)	0.122**	0.133*	0.000	0.000	0.089
	Dcc (*b*)	0.579*	0.462*	0.985*	0.613	0.603*
ΔlnGPR	Avr. Corr	0.512	0.024	0.062	0.128**	0.019
	Dcc (*a*)	0.029**	0.015	0.025	0.000	0.000*
	Dcc (*b*)	0.971*	0.944*	0.001	0.745	0.666***

Dcc (*a*) and Dcc (*b*) are the short-run and long-run DCC parameters, respectively. The symbols, ‘*,’ ‘**,’ and ‘***’ indicate the significance at 1%, 5%, and 10% levels, respectively. See Appendix (5, 6, 7, 8, 9 and 10) for more details

Furthermore, because of the oil price war between Russia and Saudi Arabia, the oil price plunged in early 2020 (Hasan et al., 2021b). On the other hand, the demand for general commodities and oil has plummeted substantially during the COVID-19 period due to imposing various restrictions on global and domestic trade activities as well as imposing lockdown measures (Ahmed & Sarkodie, 2021; Hasan et al., 2021b). As a result, the commodity markets are adversely impacted by the EPU, particularly in the COVID-19 period, consistent with Bakas and Triantafyllou (2020), Ahmed and Sarkodie (2021), Hasan et al. (2022), and Hasan et al. (2021a). Thus, investors and portfolio managers are suggested to be cautious about investing in these weak safe-haven assets—e.g., GSCI commodity and WTI—especially during the global crises.

Conversely, although the average correlation between natural gas returns and UCRY Price index changes is zero, it is more volatile during COVID-19 (Fig. 2, Panel B). Likewise, the average correlation between natural gas (soybeans) and USEPU is negative; however, from the first quarter of 2014 (2016) to the last quarter of 2016 (first quarter of 2019), the average correlation was consistently positive (Panel-C). Therefore, natural gas and soybeans were able to provide a safe-haven opportunity against USEPU during that period, supported by Badshah et al. (2019) and Ji et al. (2020). However, an extreme negative average correlation is noticed between USEPU and CE; thus, against the USEPU, CE stocks do not function as a safe-haven asset, contrasting with Haq et al. (2021), who find CE stocks as a safe haven against EPU.

Interestingly, all commodities have a positive average correlation with GPR (see Table 3). However, only natural gas, soybeans, and CE stock strongly correlate with GPR even during COVID-19 (Fig. 3, Panel-C). Thus, our findings signify that natural gas, soybeans, and CE stock serve as robust safe-haven tools against GPR, with GSCI and WTI showing weak safe-haven. During COVID-19, the GPR index declined from 138.42 points on January 01, 2020, to 64.07 points on December 01, 2020,^7^ indicating an unprecedented drop in geopolitical risk globally. However, it is worth noting that the GPR index covers only the non-financial factors (e.g., war, terror attacks, conflicts between states, etc.), which were absent during the pandemic, especially in 2020, as the whole world’s concentration turned into a global health crisis. Conversely, as mentioned above, the commodity market demand and its prices fell substantially in early 2020 because of the worldwide lockdowns due to the COVID-19. These events may be the possible reason for the positive association between GPR and commodities. Nonetheless, our findings are consistent with Ding et al. (2021) and partially with Smales (2021), but the GPR-WTI nexus contradicts Antonakakis et al. (2017) and Qin et al. (2020).

Finally, all commodity assets exhibit a negative correlation with VIX and OVX, indicating that they do not provide safe haven property (Fig. 3: Panels A and B). The VIX and OVX measure the stock market and oil market volatility, respectively. Because the stock and oil markets are vital parts of any economy, any volatility or risk in these markets may induce uncertainty in the rest of the economy, resulting in a negative demand shock in other sectors like the commodity markets (Bouri et al., 2018; Fernandes et al., 2014; Lin & Tsai, 2019). As a result, the returns on these assets are reduced, emphasizing the negative association between commodity markets and VIX and OVX.

### Results from quantile-on-quantile (QQ) regression approach

**Fig. 4:**
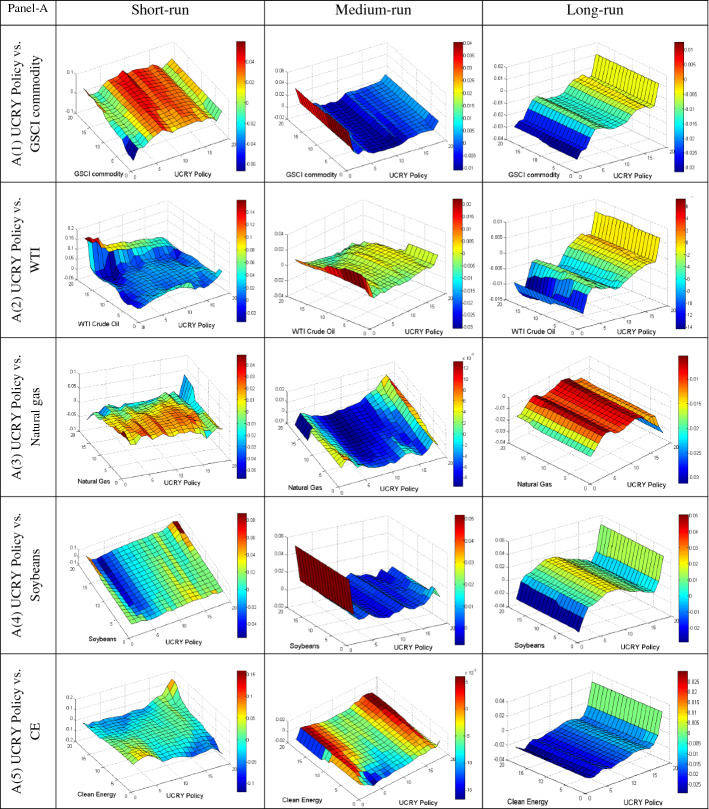

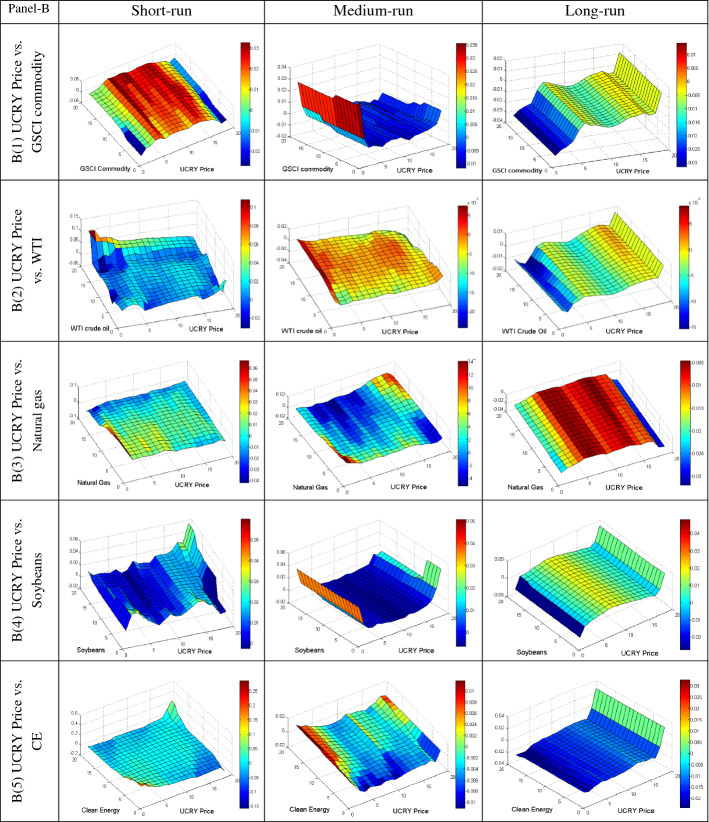

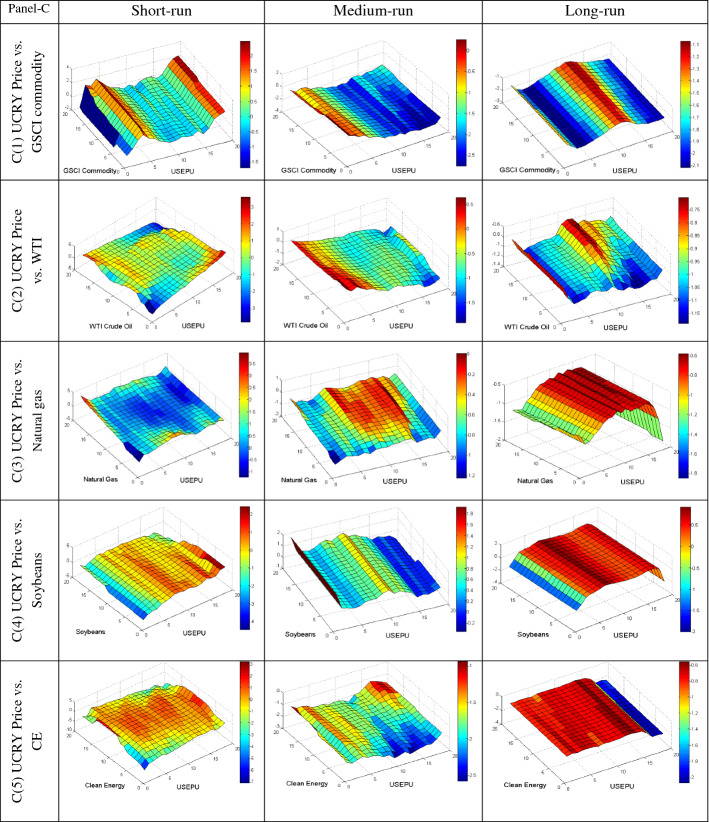

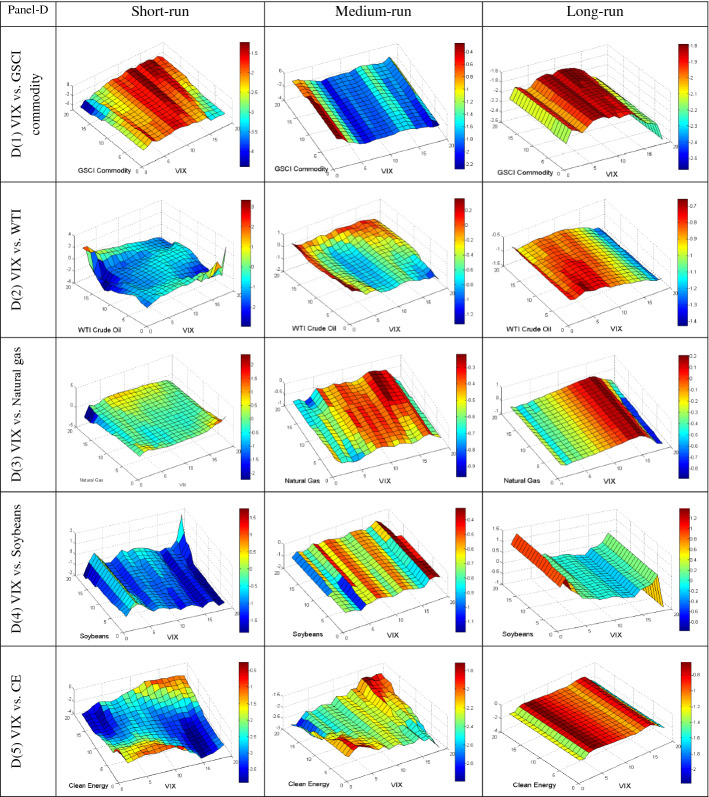

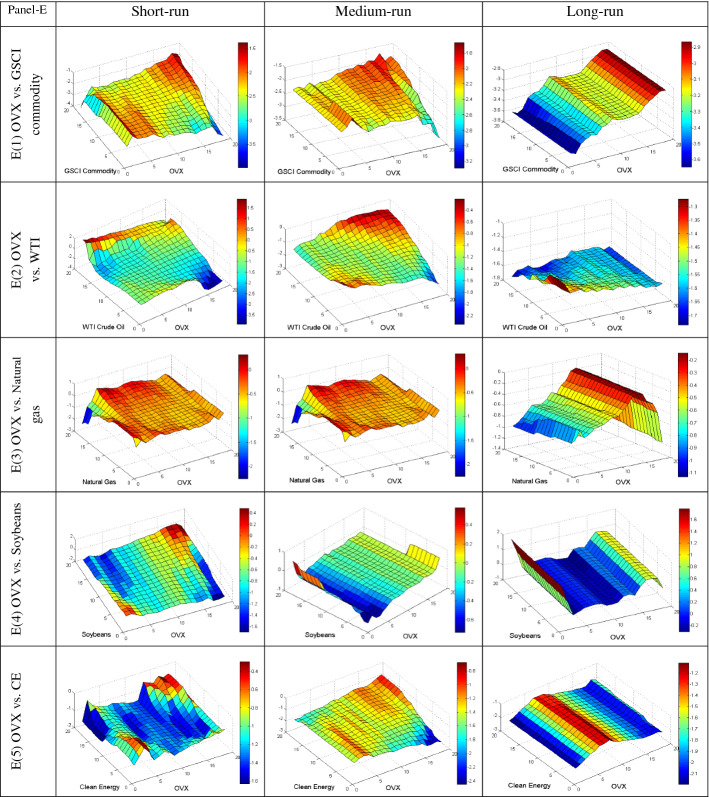

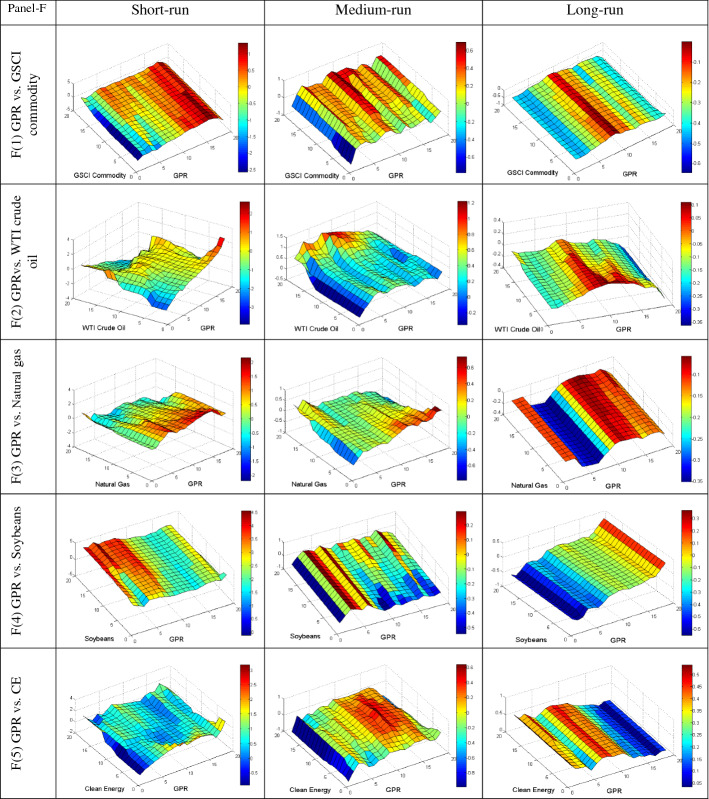
**A** Quantile-on-Quantile estimation (UCRY Policy vs. commodities). Note: The y-axis (commodities) and x-axis (uncertainties) signify the number from 1 to 19, corresponding to the quantiles from 0.05 to 0.95. **B** Quantile-on-Quantile estimation (UCRY Price vs. commodities). Note: For explanation, see Fig. 4A. **C** Quantile-on-Quantile estimation (USEPU vs. commodities). Note: For explanation, see Fig. 4A. **D** Quantile-on-Quantile estimation (VIX vs. commodities). Note: For explanation, see Fig. 4A. **E** Quantile-on-Quantile estimation (OVX vs. commodities). Note: For explanation, see Fig. 4A. **F** Quantile-on-Quantile estimation (GPR vs. commodities). Note: For explanation, see Fig. 4A

**Fig. 5:**
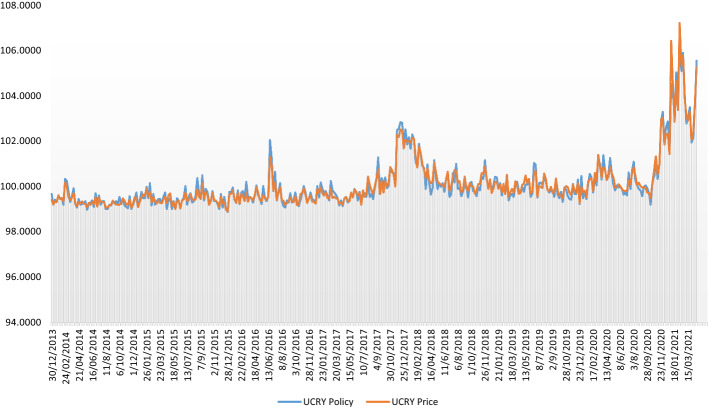
UCRY policy and price trend

We further employ the QQ regression technique to reveal the quantile’s impact of different uncertainties on the conditional distributions of commodities in various frequencies to provide more insight into their dependency. The QQ results are plotted in Fig. 4A–E, based on short, medium, and long-run phases. The individual graphs estimate the slope coefficient in the z-axis for the quantiles of the y-axis (commodities) and x-axis (uncertainties). The bullish and bearish conditions of the market are represented by upper and lower quantiles of commodities, respectively. Conversely, the upper and lower quantiles of uncertainties represent the high and low uncertainty, respectively.

The results in Fig. 4A1 show that in the short run, the lower quantiles (0.05–0.20) of UCRY Policy negatively impact the lower to upper quantiles (0.05–0.95) of GSCI commodity. Furthermore, the middle to higher quantiles (0.25–0.95) of GSCI are positively influenced by the middle to upper quantiles (0.20–0.95) of UCRY Policy. Similarly, the UCRY Policy impacts the GSCI positively only in the lower and upper quantiles in the medium-run. Conversely, the GSCI is negatively impacted until the upper quantile, focusing on the long-run impact. Thus, when the UCRY Policy is at the normal and riskier conditions, the positive response of GSCI is noticed in all market circumstances in the short run. Therefore, we observe that the GSCI commodity can provide a weak safe haven for the short and long run.

Moreover, in Fig. 4A2, the whole quantiles of the UCRY Policy positively influence the WTI when the WTI market is bullish (upper quantiles) in the short run. In the medium-run, a strong positive impact by the lower quantiles of UCRY Policy is found in the lower to upper quantiles of WTI, whereas against the long-run upper quantiles of UCRY Policy, the WTI responds negatively throughout the market conditions. Thus, against UCRY Policy, the WTI market can provide hedging opportunities in the short run. Surprisingly, the natural gas returns show a strong adverse reaction to UCRY Policy changes in all investment horizons (Fig. 4A3), suggesting that the natural gas fails to hedge UCRY Policy.

Turning to Fig. 4A4, only the upper quantiles of soybean and UCRY Policy are positively correlated in the short run. However, soybean has a continuous positive relationship with UCRY Policy from the lower to higher quantiles of both market returns in the medium-run and middle to upper quantiles in the long run. Therefore, soybean can hedge the UCRY Policy in the medium and long run. Likewise, in Fig. 4A5, lower to middle quantiles (0.05–0.50) of UCRY Policy impact positively on the lower to middle quantiles (0.05–0.75) of CE returns in the short-run. Conversely, UCRY Policy has a negative effect on the CE returns (except for lower–upper and upper quantiles of UCRY Policy and CE, respectively) in the medium-run. At the higher UCRY Policy, CE strongly mitigates UCRY Policy, reflecting the safe-haven opportunity of CE stock.

Figure 4B displays the results of UCRY Price and five commodities and exhibits that in the short-run, from lower to upper quantiles of UCRY Price has a strong positive impact on the middle quantile of GSCI. Moreover, in the medium run, the lower quantiles of UCRY Price influence the GSCI positively, while the negative impact is observed in the long run. Thus, GSCI serves as a weak hedge against UCRY Price. Likewise, in the short run, Fig. 4B2 unveils that the upper quantiles of WTI are positively affected by the whole quantile of UCRY Price. Similarly, in the medium and long run, the UCRY Price impacts the WTI negatively except for the lower quantile of UCRY Price on the middle to higher quantile of WTI in the medium run. Conversely, natural gas returns are strongly negatively impacted by the UCRY Price changes in all frequencies, although a positive relationship is observed in the upper quantile of both.

In Fig. 4B4, the whole quantiles of UCRY Price positively affect the middle to upper quantiles (0.30–0.95) of soybean returns in the short and long run. Similarly, in the mid-term, soybean responds positively except for the middle quantiles of UCRY Price. A similar result is also found in the case of CE stock, e.g., 0.35–0.95 quantiles are positively impacted by all quantiles of UCRY Price (except 0.30–0.45) in the medium-run. When the UCRY Price increases, the CE stocks also increase in the long run. Therefore, both the soybean and CE stock are strong risk diversifiers during high UCRY Price regimes, signifying their robust safe-haven properties.

Figure 4C1 shows that the lower and higher quantiles of USEPU and lower to higher quantiles of GSCI are positively associated in the short run. In the medium and long run, USEPU impacts GSCI negatively in whole quantiles. Likewise, a negative association is also noticed in Fig. 4C2 in all the cases except the positive impact in the lower quantile (0.05–0.15) of USEPU on WTI returns. Furthermore, natural gas (Fig. 4C3) is also negatively impacted by USEPU in both the medium and long run, while only in the short run the positive impact is noticed in the lower, middle, and higher quantiles of USEPU on the middle to upper, lower and upper, and lower quantiles of natural gas, respectively. Consistently, in Fig. 4C4, the middle and upper quantiles of USEPU impact positively on the lower to middle quantiles of soybean in the short run.

Finally, the lower to middle and middle to upper quantiles of USEPU have a positive impact on all quantiles of soybean in the medium- and long run, respectively, whereas the USEPU index changes in all investment horizons have a negative impact on CE stock (except the middle quantile) in the short run. Therefore, we can conclude that among the commodities, only soybean exhibit weak safe-haven properties against USEPU.

Figure 4D describes the impacts of VIX on commodities. Figure 4D1 shows that VIX negatively impacts the GSCI in both the lower and upper quantiles in all frequencies. Conversely, in the medium run, Fig. 4D2 reveals that only the lower and upper quantiles of VIX positively impact the upper quantiles of WTI returns. Figure 4D3 unveils that the upper quantiles of VIX and natural gas returns have a positive correlation both in the short and long run.

Moreover, there is a highly antagonistic relationship between VIX and soybean returns in the short- and medium-run. At the same time, only the lower and upper quantiles of VIX positively impact the lower to upper quantiles of soybean. Conversely, a strong negative relation is found between VIX and CE stock. Overall, VIX and almost all commodity assets are negatively correlated, indicating that commodity assets can not hedge the VIX shocks.

Figure 4E exhibits that all the commodities (except soybean) are negatively affected by OVX in the entire frequencies. Figure 4E4 shows a positive relationship between the lower and upper quantiles of OVX and soybean in the short run. Similarly, a positive association exists in the lower–upper and upper-lower quantiles of OVX and soybeans in the medium run, respectively. In the long run, the lower and higher quantiles of OVX positively impact the bearish and bullish conditions of the soybean market. Therefore, only soybeans can weakly hedge OVX.

Finally, Fig. 4F shows a positive association between GPR and the commodity assets except for some quantiles. Notably, at a time of highly bullish uncertainty, soybean and CE stock show a strong positive correlation across all the frequencies. Thus, we can conclude that soybeans and CE stock can function as robust safe-haven tools. Conversely, GSCI has a positive association except for lower quantile in the medium run with GPR across the quantiles and middle quantile in the medium and long runs, respectively. Similarly, (somewhat surprisingly) the natural gas responds positively when the uncertainty is extreme for all the quantiles, while the reverse is valid for WTI. Therefore, GSCI and natural gas may weakly diversify the risk derived from GPR.

Overall, except for natural gas, all commodities are positively linked with the UCRY Policy and Price and GPR during COVID-19. According to the summary in Table 4, our findings also reveal that all commodities (excluding the soybean) are negatively linked with VIX, as well as the USEPU and OVX. Furthermore, the strong (i.e., soybean and CE stocks) or weak (i.e., GSCI, WTI oil, and CE stock) safe-haven assets against cryptocurrency uncertainty are adversely related to other uncertainty indices (USEPU, VIX, and OVX), except GPR. As a result, we can conclude that both of the cryptocurrency uncertainty indices impact commodities differently from other uncertainty and volatility indices, excluding GPR, hence supporting the hypothesis of Lucey et al. (2022). Also, our findings indicate that the safe-haven properties of commodity assets alter across different frequencies and quantiles.

**Table 4:** Safe-haven summary from the QQ estimations

Safe-haven candidates	Uncertainty indices											
	UCRY Policy		UCRY Price		USEPU		VIX		OVX		GPR	
	Weak	Strong	Weak	Strong	Weak	Strong	Weak	Strong	Weak	Strong	Weak	Strong
GSCI commodity	**√**		**√**								**√**	
WTI	**√**		**√**									
Natural gas											**√**	
Soybeans	**√**			**√**	**√**				**√**			**√**
CE	**√**			**√**								**√**

“√” represents the presence of safe-haven properties

Although the cryptocurrency markets are growing fast, their market size is still fairly small compared to the stock and oil markets and the overall economy. Cryptocurrencies represent the new digital form of currency, and they are not massively used as mediums of exchange worldwide yet. Hence, it is likely to have a comparatively lower influence on other markets. On the other hand, the commodity markets are more relevant to aggregate economic activities, stock markets, and oil markets than cryptocurrency markets. Therefore, any uncertainty from the economy, stock markets, and oil markets tend to have a more adverse influence on the commodity markets than the cryptocurrency markets.

## Conclusions

This study analyses the hedging and safe-haven features of five major commodity assets against six popular uncertainty indicators. Employing the DCC-GJR-GARCH (1, 1) and Wavelet-based Quantile-on-Quantile techniques on the weekly observations from December 30, 2013, to April 22, 2021, we show that only soybean and clean energy stock markets might provide a strong hedge against UCRY Price and GPR, including during COVID-19. We find poor safe-haven properties in the GSCI commodity against cryptocurrency uncertainties and GPR, WTI against cryptocurrency uncertainties, natural gas against GPR, soybean against UCRY Policy, USEPU, and OVX, and CE stock against UCRY Policy. Furthermore, our findings suggest that assets’ safe-haven features vary throughout frequencies and quantiles. Finally, except for GPR, both cryptocurrency uncertainty indices impact commodities in the opposite direction of other uncertainty and risk indices.

Our findings contribute significantly to the investor’s portfolio management strategies in several ways. First, investors and portfolio managers may use our findings to better forecast the risks and impacts of different uncertainties on their portfolios and identify a notable collection of alternative assets to diversify their portfolios against such uncertainties. Second, investors and policymakers may better understand the risks associated with the cryptocurrency market and its impact on commodity markets. Third, our findings may be of great interest when investors look for risk diversifiers during crises, particularly COVID-19-like global shock episodes. To be specific, our result suggests that investors might use commodity assets such as soybeans and clean energy stocks to hedge uncertainties and risks derived from the cryptocurrency market and geopolitical events. Furthermore, because WTI crude oil and GSCI commodity are proven to be weak risk diversifiers, we recommend that investors should become more cautious about investing in them. Fourth, our findings urge commodity market policymakers—mainly GSCI, WTI crude oil, natural gas, and clean energy stocks—to be aware of the adverse effects of the vulnerability of US economic policy, stock market volatility, and crude oil volatility. Finally, our study suggests that the commodity investors should consider the digital currency market uncertainties differently since the UCRY indices show a distinct impact pattern than other uncertainty indices.

Future research can be conducted by, e.g., incorporating more commodity assets or other financial assets and scrutinizing the effects of various uncertainty indicators, including macroeconomic uncertainty, monetary policy uncertainty, etc., on such assets' risk/return profile. Another limitation of this study is that it could not consider the recent Russia-Ukraine war issue, although the phenomenon has created havoc in the commodity markets. However, it is too early to measure the accurate impact of the war. Hence, further studies are encouraged to incorporate this issue to assess the war’s precise impact on the commodity markets. Future research should also focus more profoundly on, e.g., the behavioral patterns in cryptocurrency or other uncertainty indicators.

## Data Availability

The data sources are given in the data and methodology section of the paper. The datasets are provided on reasonable request.

## Appendix

See Fig. 5 and Tables 5, 6, 7, 8, 9 and 10.

**Table 5:** Results from DCC-GJR-GARCH (1, 1) estimation (UCRY Policy and commodities)

	ΔlnUCRYPolicy	ΔlnGSCI	ΔlnUCRY Policy	ΔlnWTI	ΔlnUCRY Policy	ΔlnNatural gas	ΔlnUCRY Policy	ΔlnSoybeans	ΔlnUCRY Policy	ΔlnCE
*Panel A: AR (1)-GARCH (1, 1) estimation*										
Const. (M)	0.000**	− 0.000	0.000**	− 0.003	0.000**	0.001	0.000**	0.001	0.000**	0.003***
AR (1)	− 0.458*	0.136**	− 0.458*	0.096***	− 0.458*	0.170*	− 0.458*	0.128**	− 0.458*	0.040
Const. (V)	0.717**	0.856*	0.717**	4.254*	0.717**	1.279***	0.717**	1.148	0.717**	0.587
⍺ (ARCH 1)	0.244*	− 0.065**	0.244*	− 0.100**	0.244*	0.182*	0.244*	0.087	0.244*	0.219*
β (GARCH 1)	0.745*	0.788*	0.745*	0.684*	0.745*	0.820*	0.745*	0.730	0.745*	0.762*
(⍺ + β)	0.989	0.723	0.989	0.584	0.989	1.002	0.989	0.817	0.989	0.981
GJR(Gamma)	− 0.225***	0.307*	− 0.225***	0.454*	− 0.225***	− 0.166***	− 0.225***	− 0.038	− 0.225***	0.023
*Panel B: Diagnostic tests*										
Qs (20)	39.323*	15.301**	39.026	28.072	29.783	26.580	32.827	11.772	32.509	14.803
Hosking (20)	122.452*		122.404		91.289		86.275		81.807	
Li– McLeod(20)	121.784*		121.798		91.445		86.728		82.291	
*Panel C: Information criteria*										
Akaike	− 12.702		− 11.577		− 11.431		− 12.918		− 12.386	
Shibata	12.705		− 11.581		− 11.435		− 12.922		− 12.389	
Hannan– Quin	− 12.636		− 11.512		− 11.366		− 12.853		− 12.320	

Qs (20) for Ljung–Box test statistics, Hosking and McLeod, and Li are multivariate Portmanteau statistics for checking the null hypothesis of no serial correlation (using 20 lags). ∗ , ∗  ∗ , and ∗  ∗  ∗ indicate the significance at 1%, 5%, and 10% levels, respectively

**Table 6:** Results of DCC-GJR-GARCH (1, 1) estimation (UCRY Price and commodities)

	ΔlnUCRY Price	ΔlnGSCI	ΔlnUCRY Price	ΔlnWTI	ΔlnUCRY Price	ΔlnNatural gas	ΔlnUCRY Price	ΔlnSoybeans	ΔlnUCRY Price	ΔlnCE
*Panel A: AR (1)-GARCH (1, 1) estimation*										
Const. (M)	0.000**	− 0.000	0.000**	− 0.003	0.000**	0.001	0.000**	0.001	0.000**	0.003***
AR (1)	− 0.451*	0.136**	− 0.451*	0.096***	− 0.451*	0.170*	− 0.451*	0.128**	− 0.451*	0.040
Const. (V)	0.359	0.856*	0.359	4.254*	0.359	1.279***	0.359	1.148	0.359	0.587
*⍺* (ARCH 1)	0.275*	− 0.065***	0.275*	− 0.100**	0.275*	0.182*	0.285*	0.087	0.285*	0.219*
*β* (GARCH 1)	0.769*	0.788*	0.769*	0.684*	0.769*	0.820*	0.769*	0.730	0.769*	0.762*
(⍺ + β)	1.001	0.723	1.001	0.584	1.001	1.002	1.001	0.817	1.001	0.981
GJR(Gamma)	− 0.320**	0.307*	− 0.320**	0.454*	− 0.320**	− 0.166***	− 0.320**	− 0.038	− 0.320**	0.023
*Panel B: Diagnostic tests*										
Qs (20)	34.862**	33.294**	30.853	28.023	24.210	26.578	29.431	11.735	29.465	14.811
Hosking (20)	105.254**		96.422		90.002		76.868		81.113	
li– McLeod (20)	104.946**		101.062		90.066		77.403		81.427	
*Panel C: Information criteria*										
Akaike	− 13.155		− 12.033		− 11.880		− 13.374		− 12.849	
Shibata	− 13.158		− 12.036		− 11.884		− 13.377		12.853	
Hannan– Quinn	− 13.089		− 11.968		− 11.815		− 13.308		− 12.784	

Please see the notes in Fig. 5

**Table 7:** Results of DCC-GJR-GARCH (1, 1) estimation (USEPU and commodities)

	ΔlnUSEPU	ΔlnGSCI	ΔlnUSEPU	ΔlnWTI	ΔlnUSEPU	ΔlnNatural gas	ΔlnUSEPU	ΔlnSoybeans	ΔlnUSEPU	ΔlnCE
*Panel A: AR (1)-GARCH (1, 1) estimation*										
Const. (M)	− 0.008	− 0.000	− 0.008	− 0.003	− 0.008	0.001	− 0.008	0.001	− 0.008	0.003***
AR (1)	− 0.343*	0.136**	− 0.343*	0.096***	− 0.343*	0.170*	− 0.343*	0.128**	− 0.343*	0.040
Const. (V)	0.049*	0.856*	0.049*	4.254*	0.049*	1.279***	0.049*	1.148	0.049*	0.587
*⍺* (ARCH 1)	0.138***	− 0.065***	0.138***	− 0.100**	0.138***	0.182*	0.138***	0.087	0.138***	0.219*
*β* (GARCH 1)	0.300**	0.788*	0.300**	0.684*	0.300**	0.820*	0.300**	0.730	0.300**	0.762*
(*⍺* + *β*)	0.438	0.723	0.438	0.584	0.438	1.002	0.438	0.817	0.438	0.981
GJR(Gamma)	0.149	0.307*	0.149	0.454*	0.149	− 0.166***	0.149	− 0.038	0.149	0.023
*Panel B: Diagnostic tests*										
Qs (20)	22.920	25.667	26.274	28.579	27.612	26.562	25.665	11.793	34.845	14.627
Hosking (20)	107.659		102.275		101.274		103.220		100.530	
li– McLeod (20)	127.267		131.703		110.948		112.992		113.319	
*Panel C: Information criteria*										
Akaike	− 3.956		− 2.820		− 2.669		− 4.179		− 3.611	
Shibata	− 3.959		− 2.823		− 2.673		− 4.183		− 3.614	
Hannan– Quinn	− 3.890		− 2.755		− 2.604		− 4.114		− 3.545	

Please see the notes in Fig. 5

**Table 8:** Results of DCC-GJR-GARCH (1, 1) estimation (VIX and commodities)

	ΔlnVIX	ΔlnGSCI	ΔlnVIX	ΔlnWTI	ΔlnVIX	ΔlnNatural gas	ΔlnVIX	ΔlnSoybeans	ΔlnVIX	ΔlnCE
*Panel A: AR (1)-GARCH (1, 1) estimation*										
Const. (M)	0.007	− 0.000	0.007	− 0.003	0.007	0.001	0.007	0.001	0.007	0.003***
AR (1)	− 0.160	0.136**	− 0.160	0.096***	− 0.160	0.170*	− 0.160	0.128**	− 0.160	0.040
Const. (V)	0.009**	0.856*	0.009**	4.254*	0.009**	1.279***	0.009**	1.148	0.009**	0.587
*⍺* (ARCH 1)	0.381***	− 0.065***	0.381***	− 0.100**	0.381***	0.182*	0.381***	0.087	0.381***	0.219*
*β* (GARCH 1)	0.549*	0.788*	0.549*	0.684*	0.549*	0.820*	0.549*	0.730	0.549*	0.762*
(*⍺* + *β*)	0.930	0.723	0.930	0.584	0.930	1.002	0.930	0.817	0.930	0.981
GJR(Gamma)	− 0.508**	0.307*	− 0.508**	0.454*	− 0.508**	− 0.166***	− 0.508**	− 0.038	− 0.508**	0.023
*Panel B: Diagnostic tests*										
Qs (20)	29.678	24.161	29.925	25.250	29.726	26.503	29.930	13.220	28.800	20.349
Hosking (20)	87.515		94.671		93.374		86.078		83.530	
li– McLeod (20)	87.271		94.163		99.673		85.841		83.454	
*Panel C: Information criteria*										
Akaike	− 5.404		− 4.263		− 4.039		− 5.544		− 5.306	
Shibata	− 5.408		− 4.266		− 4.043		− 5.547		− 5.309	
Hannan– Quinn	− 5.339		− 4.197		− 3.974		− 5.479		− 5.240	

Please see the notes in Fig. 5

**Table 9:** Results of DCC-GJR-GARCH (1, 1) estimation (OVX and commodities)

	ΔlnOVX	ΔlnGSCI	ΔlnOVX	ΔlnWTI	ΔlnOVX	ΔlnNatural gas	ΔlnOVX	ΔlnSoybeans	ΔlnOVX	ΔlnCE
*Panel A: AR (1)-GARCH (1, 1) estimation*										
Const. (M)	0.003	− 0.000	0.003	− 0.003	0.003	0.001	0.003	0.001	0.003	0.003***
AR (1)	− 0.146**	0.136**	− 0.146**	0.096***	− 0.146**	0.170*	− 0.146**	0.128**	− 0.146**	0.040
Const. (V)	0.002*	0.856*	0.002*	4.254*	0.002*	1.279***	0.002*	1.148	0.002*	0.587
*⍺* (ARCH 1)	0.272*	− 0.065***	0.272*	− 0.100**	0.272*	0.182*	0.272*	0.087	0.272*	0.219*
*β* (GARCH 1)	0.710*	0.788*	0.710*	0.684*	0.710*	0.820*	0.710*	0.730	0.710*	0.762*
(*⍺* + *β*)	0.982	0.723	0.982	0.584	0.982	1.002	0.982	0.817	0.982	0.981
GJR(Gamma)	− 0.299**	0.307*	− 0.299**	0.454*	− 0.299**	− 0.166***	− 0.299**	− 0.038	− 0.299**	0.023
*Panel B: Diagnostic tests*										
Qs (20)	28.399	28.111	29.067	25.420	29.865	24.746	24.260	11.972	30.432	15.027
Hosking (20)	97.392		91.69		101.350		71.215		93.769	
li– McLeod (20)	96.798		91.322		103.815		71.332		93.311	
*Panel C: Information criteria*										
Akaike	− 6.329		− 5.247		− 4.740		− 6.266		− 5.841	
Shibata	− 6.332		− 5.250		− 4.743		− 6.269		− 5.844	
Hannan– Quinn	− 6.263		− 5.181		− 4.675		− 6.200		− 5.775	

Please see the notes in Fig. 5

**Table 10:** Results of DCC-GJR-GARCH (1, 1) estimation (GPR and commodities)

	ΔlnGPR	ΔlnGSCI	ΔlnGPR	ΔlnWTI	ΔlnGPR	ΔlnNatural gas	ΔlnGPR	ΔlnSoybeans	ΔlnGPR	ΔlnCE
*Panel A: AR (1)– GARCH (1, 1) estimation*										
Const. (M)	0.016	− 0.000	0.016	− 0.003	0.016	0.001	0.016	0.001	0.016	0.003***
AR (1)	− 0.154	0.136**	− 0.154	0.096***	− 0.154	0.170*	− 0.154	0.128**	− 0.154	0.040
Const. (V)	0.026**	0.856*	0.026**	4.254*	0.026**	1.279***	0.026**	1.148	0.026**	0.587
*⍺* (ARCH 1)	0.163***	− 0.065***	0.163***	− 0.100**	0.163***	0.182*	0.163***	0.087	0.163***	0.219*
*β* (GARCH 1)	0.508***	0.788*	0.508***	0.684*	0.508***	0.820*	0.508***	0.730	0.508***	0.762*
(*⍺* + *β*)	0.671	0.723	0.671	0.584	0.671	1.002	0.671	0.817	0.671	0.981
GJR(Gamma)	− 0.227*	0.307*	− 0.227*	0.454*	− 0.227*	− 0.166***	− 0.227*	− 0.038	− 0.227*	0.023
*Panel B: Diagnostic tests*										
Qs (20)	65.343*	29.787***	65.168*	32.082**	65.231*	41.087*	65.001*	20.111	65.069*	14.239*
Hosking (20)	137.577*		140.197*		149.910*		122.217*		113.802*	
li– McLeod (20)	137.177*		139.668*		148.787*		122.216*		113.801*	
*Panel C: Information criteria*										
Akaike	− 4.441		− 3.310		− 3.252		− 4.671		− 4.163	
Shibata	− 4.444		− 3.312		− 3.254		− 4.673		− 4.166	
Hannan– Quinn	− 4.384		− 3.252		− 3.194		− 4.613		− 4.106	

Please see the notes in Fig. 5

## Footnotes

1 UCRY Policy and Price indices are introduced to capture the uncertainties arising from the cryptocurrency markets. For more details concerning such indices, see Lucey et al. (2022).

2 For keeping conformity of the sample range of each variable, we use weekly data and the sample period, which forces us to abandon some important uncertainty measures, particularly, macroeconomic and global financial uncertainty.

3 The starting of COVID-19 period is selected based on the outbreak of coronavirus disease that was first reported in Wuhan, China, on 31 December 2019 (Hasan et al., 2021b).

4 The GJR-GARCH (1, 1) model, i.e., the representation with one lag for both the variance and squared residual terms in the GARCH-part is selected based on the Akaike Information Criterion (AIC) and Schwarz Criterion (SC).

5 Details of the wavelet-based decomposition calculation are available upon request.

6 https://www.bloomberg.com/news/articles/2021-01-29/bitcoin-investors-may-lose-everything-central-banker-warns.

7 https://www.matteoiacoviello.com/gpr.htm
